# Developing hospital resilience domains in facing disruption era in Indonesia: a qualitative study

**DOI:** 10.1186/s12913-023-10416-8

**Published:** 2023-12-12

**Authors:** Nurmala Sari, Maye Omar, Syahrir A. Pasinringi, Andi Zulkifli, Andi Indahwaty Sidin

**Affiliations:** 1 Hospital Management Department, Public Health Faculty, Hasanuddin University, Makassar, Indonesia; 2 Nuffield Centre for International Health and Development, Leeds Institute of Health Sciences, School of Medicine, University of Leeds, Leeds, UK; 3 Magister of Hospital Administration Study Program, Public Health Faculty, Hasanuddin University, Makassar, Indonesia

**Keywords:** Disasters, Disruption, Domains, Hospitals, Resilience

## Abstract

**Background:**

The studies of hospital resilience have been of increasing importance during the last decade due to disasters and pandemics. However, studies in developing the domain and indicators of hospital resilience were limited mainly on disaster response. A few studies of hospital resilience focused on how to deal with disruptions such as environmental turbulence, rapid technological changes, and changes in patient preferences. This study aims to develop domains and indicators of hospital resilience in facing the disruption era.

**Methods:**

This qualitative study focused on exploring the domains and indicators to face disruptions that have been identified in the first exploratory phase of the studies. Key informants included hospital experts from the government, medical practitioners, and academics. A total of 20 key informants were involved in semi-structured interviews which were conducted face-to-face, via telephone and Zoom. Data was analyzed using a grounded theory approach to discover domains for a resilient hospital.

**Results:**

The study identified a number of domains that are fundamental for a hospital to become a resilient in the face of disruption. These include readiness to face digital transformation, effective leadership, and flexibility in managing resources among others. Situation awareness and resilience ethos, implementation of marketing management, networking, and disaster anticipation are found to be equally important. These domains focused on the hospital’s ability to deal with specific shocks from different perspectives as the result of changes from disruptions which are inevitable within the organizational business environment.

**Conclusions:**

The domains identified in the study are able to respond to the limitations of the concept of hospital resilience, which is currently more focused on hospital disaster resilience. They can be used to measure hospital resilience in the context of the volatility, uncertainty, complexity, and ambiguity (VUCA), which are relevant to the context of the Indonesia hospital industry.

**Supplementary Information:**

The online version contains supplementary material available at 10.1186/s12913-023-10416-8.

## Introduction

The concept of hospital resilience has gained popularity due to the high number of health crises caused by natural and non-natural disasters in the past decade [1–5]. Hospital resilience reflects organizational capacity to deal with sudden changes or shocks by absorbing, adapting, or transforming their systems to remain functioning [6]. Conventionally, the hospital resilience concept was developed from traditional safety management concepts and resilience engineering [7]. The idea was discussed in Hyogo, Japan, at the World Conference on Disaster Reduction in 2005. It concerned the role of health services in disaster risk reduction and capacity building in disaster management [8].

During the last decades, resilience concepts in health studies have focused on how to develop domains and indicators [9–13] and how the concept is implemented at the national [5, 7, 13–18], organizational [12, 19–22], and individual levels [23–26]. Several resilience frameworks that have been developed by the WHO and PAHO, for example, are very specific for certain disasters, such as resilience for earthquakes, floods, climate change, and infectious disease outbreaks [4–6]. Barbash and Jeremy (2021) stated that the concept of hospital resilience, which focuses on disaster preparedness, needs to shift to a more comprehensive concept that can accommodate any challenges other than disasters [27]. Hospitals as organizations will continuously face disruptions in their business environment [28]. Brendan and Orin (2020) argue that disruption is a condition that changes the current system so that aspects within the system cannot adapt to that change [28].

Disruption in health care could include technological, economic, and societal changes [28, 29]. Additional disruptions could be policy changes [30–32], terrorism [33], and political crises [29]. Consequently, hospitals are required to be able to adapt to all these uncertain, ambiguous, and complex conditions [34]. Hospitals with resilience capabilities face disruption by considering multiple solutions to every problem and quickly maneuvering when previously planned strategies do not work [9, 27]. Resilience is more than just a contingency plan. Resilience is a way of being, a mindset, a set of skills, and a specific process that enables an organization to embrace uncertainty, survive, and recover from setbacks [35].

To date, comprehensive hospital resilience concepts are still being developed. In 2022, the WHO developed a toolkit regarding health system resilience in various contexts, including technical resources of resilience concepts at the operational level [36]. Most of the resilience health care documents identified in the toolkit are guidance that is applied in public health emergencies [37, 38], climate changes [39], the Covid-19 pandemic [40, 41], and zoonotic disease [42]. Those concepts were developed in specific contexts and written in different documents. Few hospital resilience concepts account for shocks or disruptions in highly dynamic environments.

## Resilience and the need for comprehensive hospital resilience concept

Resilience concept has been used across a number of disciplinary fields [43–46] and has been implemented and represented in different ways. Resilience in engineering perspective, primarily adopted in safety studies, focuses on the “bounce back” ability after disruption [46]. From a psychological perspective, resilience is an individual capacity to cope with traumas or challenges [47], and the ecological perspective shows resilience as how the biological system adapts and maintains its system in facing threats [47]. From all disciplinary fields, recent literature highlighted the resilience concept more comprehensively to include the ability of individuals, organizations, communities, or systems to face and cope with pressures, failures, errors, and disruptions by means of flexibility and reorganizing or enhancing their capacities to return to normal function [48].

The resilience concept has been implemented at each level of healthcare system in the health sector, particularly in hospitals. Studies of hospital resilience as an organization has developed domains and indicators to measure hospital resilience [20, 49–52]. However, the domains and indicators developed are mainly about the readiness of the input aspects of the hospital, such as structural, non-structural, and specific disaster organizational functions. In response to the need for the hospital resilience concept in facing any disruption at the organizational level in health care. In this study, we adopted the organizational resilience theory developed in business and management literature, which focuses on a dynamic perspective.

In business and management literature, there are two views of organizational resilience theory, namely static perspective [53–56] and dynamic perspective [26, 57–66]. The static perspective views resilience as a function and resilience focusing on the availability of inputs. It compares organization’s conditions before and after a crisis. In contrast, the dynamic perspective views resilience as a capacity and a process where resilience is seen as the spirit of an organization that should always be present in the organization’s daily routines to be ready to face any challenge, whether expected or unexpected [67, 68]. In the Health sector, as the domain of organizational resilience focuses on preparedness that is mostly about the inputs or to prepare the hospital to remain functioning, it can be seen that the organizational resilience view adopted still static perspective. Hospitals, as organizations with complex functions, also will face dynamic situations. There is a need to develop a resilience concept from a dynamic perspective that focuses on capacity and process.

## Overview of Hospital Industries in Indonesia

Indonesia has a healthcare system comprising public and private providers. The public system in Indonesia follows the decentralized governance system, where the responsibilities are divided among the central, provincial, and district governments [69]. There are three types of healthcare services in Indonesia, namely primary, secondary, and tertiary. Primary healthcare services provide basic preventive and curatice services through community health centers and clinics. Secondary healthcare services are intended for individuals who require outpatient or inpatient care that are provided by type C hospitals and type D hospitals. Tertiary healthcare services are provided by type A and type B hospitals to include both broad and priority specialist care by specialist doctors [70]. In January 2014, the Government of Indonesia launched a social insurance plan called Jaminan Kesehatan Nasional (JKN), which was implemented by BPJS Kesehatan involving financial contributions from members and the government. Primary care providers receive payments through capitations, while hospitals are reimbursed based on episodes of service payments using INA-CBGs [69].

The development of the hospital industry in Indonesia has accelerated over the past decade. In 2021, the total number of hospitals in Indonesia increased by 169.73% compared to 2011. Based on ownership, private hospitals in Indonesia amounted to 1496 units out of 2522 units in 2021 [71]. This number has increased by 83.55% since 2012, when there were only 815 units [70]. Hospitals in Indonesia are categorized based on ownership and class. Based on the ownership, there are public and private hospital [72]. Public hospitals consist of hospitals owned by the Ministry of Health, provincial governments, district/city governments, the Indonesian National Army, the Indonesian National Police, or other ministries. Private hospitals are owned by foundations, companies, or investors (domestic and foreign). Based on the hospital classification, the hospitals are categorized into hospitals type A (> 250 beds), type B (200- < 250 beds), type C (100- < 200 beds) and type D (50- < 100 beds) [73].

The COVID-19 pandemic has made all stakeholders in the health sector realize that health resilience policies are a priority issue, so the concept of resilience becomes one of the six pillars of the transformation of the health system discussed in the Minister of Health’s Regulation No. 13 of 2022 regarding the Strategic Plan of the Ministry of Health for the year 2020–2024. Unfortunately, the transformation of the health resilience system in this policy only discusses improving the health system’s resilience for the pharmaceutical and medical device sectors. This policy does not address the role of hospitals in the context of disruptions era [74].

## Background

In 2020, the COVID-19 pandemic in Indonesia became a turning point in adapting the concept of resilience. Hospital services struggled to keep functioning during the pandemic. At the same time, various policies for organizing hospitals in a new normal way, the growth of online start-up health consulting, digitization in health care, consumer behavior changes, and improvement of hospital quality standards were established. These responses to the pandemic changed the landscape of the healthcare in the country in general and hospital industry in particular.

Before the COVID-19 pandemic, the hospital resilience concept was recognized as an indicator in The National Hospital Accreditation Standard Edition-1 [75]. It was stated in the ‘Facilities and Health Management standard; which discussed how to be a safe hospital by applying the Hospital Safety Index (HSI) measurement once every year [76]. The Hospital Safety Index is a tool for self-assessment to measure a hospital’s readiness and resilience so that a hospital will remain operational in emergencies and disasters [77]. This traditional concept of resilience was adopted from safety management strategies. The domains and indicators of hospital resilience used are similar to the concept in Hospital Disaster Resilience (HDR) studies focusing on disaster preparedness [78–80].

Based on national hospital accreditation data in 2022, of the 3072 hospitals in the country, 78% of them received full accreditation, while the remaining 22% are not accredited [81]. This represents the number of hospitals that have not implemented the Hospital Safety Index (HSI) self-assessment possibly due to lack for conducting assessments that can describe a hospital’s readiness to prepare for disasters. A study conducted by Sunindijo et al. (2020) found that the level of HSI achievement in West Java is only 0.553 and in Yogyakarta, it is 0.527 (Level B),. Even In Yogyakarta, the achievement of the disaster and emergency management module is only 0.305 (level C) [82]. These findings indicate the need for intervention measures to address the issue.

It is important to acknowledge the fact that one of the weaknesses of using HSI to measure hospital resilience is that this tool is only used to deal with emergencies and disaster shock conditions [83]. At the time of COVID-19 pandemic, hospitals in Indonesia faced a significant increase in demand for COVID-19 services [84], a decrease in non-Covid-19 service utilities [85], allocation of healthcare spending to mobilize sufficient resources to combat the pandemic [86], and changes in society’s behavior in accessing healthcare services [85, 87, 88].

Experience in the COVID-19 pandemic showed the need for health system transformation even at the hospital service level. The world is rapidly evolving, creating a sense of urgency for organizations to adapt for their sustainability. This change has been labeled as VUCA (Volatility, Uncertainty, Complexity, and Ambiguity), referring to the future world on the brink of transformation [89]. The healthcare industry has experienced the most VUCA times. The COVID-19 pandemic has presented unique challenges to leadership teams, resulting in the need for significant changes to their practices [89].

Due to health system transformation policies that focus on the agenda of strengthening hospital resilience. The substantial changes in the landscape of the hospital industry in Indonesia could not be accommodated by the Hospital Safety Index Concept. There is a need to develop comprehensive hospital resilience that focuses on organizational processes that can face the complexity of disruption in the health care system. This study is a part of the exploratory phase of Hospital Resilience which aims to identify the domains and indicators to tackle the shocks identified in the first phase of the study.

## Methods

This current study is the second phase of the Conceptual Model of Hospital Resilience study. Interviews are conducted in two phases. The first phase was to explore the shocks that hospitals face in the disruption era in the context of the Indonesian hospital industry. The results of this phase are presented in a separate manuscript that is submitted for publication. The second phase consists of interviews regarding capacities needed to tackle the shocks that are reported by informants in the first phase, which focused on capacities to be resilient hospitals. The interviews of two phases of studies were conducted using interview questionnaire that is provided as a supplementary file (Supplementary file 1).

This qualitative study was conducted in Makassar, South Sulawesi Province, Indonesia. Key informants were identified from some provinces of Indonesia who attended the National Seminar of Hospital Accreditation held in August 2022 at Claro Hotel Makassar. Informants in this study were experts in hospital industries or resilience sciences and included policymakers from the Ministry of Health, healthcare practitioners, hospital management practitioners, and academics. The inclusion criteria were having experience of more than 5 years in the field and being willing to take part in an interview. We identified 27 potential informants. 20 informants have confirmed to take part in the study. The first author contacted them to confirm their time and method (face-to-face/virtually) as preferred by informants. 15 of the 20 informants were interviewed by face to face. The remaining five informants were interviewed over the phone or via Zoom. Interviews lasted from 45 to 120 minutes.

Interviews were conducted using semi-structured questionnaire guide. All informants received an explanation letter about the study and filled out informed consent before the interview began. All interviews were conducted in Indonesian by the first author from August – November 2022 using audio recorders. This research was carried out in accordance with ethical clearance approved by the Ethics Council under number 9860/UN4.14.1/TP.01.02/2022.

This study used the grounded theory approach to discover resilience domain capacities. The analysis began with data reading and memos writing by forming a coding scheme to explore data, identify links between themes, and develop broader categories of themes. The first author made a transcript of the data soon after the first interview was completed. The transcription process was conducted while data were still being collected. Each transcription was labeled with a unique identifier as interview number (1.1–1.20). After that, we developed initial codes to identify issues raised by interviewers. We coded hospital capacities mentioned by interviewers by identifying verb words and nouns that describe hospital capacities. After completing the initial coding, the first author discussed them in a regular meeting. This was followed by focused coding. The initial codes were summarised in notes to enable the first author to categorize the focused coding. All process during initial codes and focused codes was written in memos. Regular meetings to compare data with codes, codes with codes, codes with categories, and analyzed with relevant theory and previous research were conducted during the analysis. After regular meetings, categories were grouped into domain and subdomain of hospital resilience capacities.

## Results

Table 1 shows the characteristics of the 20 informants. Thirteen (65%) of informants ​​were men, 8 (40%) were between 50 and 59 years old, 12 (60%) were hospital practitioners, 11 (55%) had master’s education, and 17 (85%) had experience of more than 10 years in their field.

**Table 1:** Characteristics of Interviewees

Variable	Interviewees	
	N	%
Sex		
Male	13	65
Female	7	35
Age		
40–49	5	25
50–59	8	40
60–69	7	35
Expertise Category		
Government	3	15
Practician	12	60
Academician	5	25
Education		
Bachelors	0	0
Masters	11	55
Doctoral	9	45
Experiences (Year)		
5–10	3	15
> 10	17	85

Based on interviews, seven domains and 33 indicators to measure hospital resilience in facing the era of disruption were identified. Table 2 shows an example of coding that explains each indicator in the domain. The identified domains were:

1. Hospital resources readiness in facing digital transformation
2. Effective leadership
3. Flexibility in managing existing resources
4. Situation awareness and resilience ethos
5. Implementation of marketing management
6. Networking
7. Disaster anticipation

**Table 2:** Domains, Indicators, and Examples of Codes

Domain	Indicators	Example of Codes
Hospital resources readiness in facing digital transformation (I.3, I.7, I.5, I.11, I.13)	Capacity building of human resources for health digitalization	The problem faced by our hospitals is human resources. It has to increase its capacity, its ability to be able to use digital electronics (I.13)
	Increasing the capacity of information technology that facilitates decision-making	Information technology must be updated, starting from all kinds of medical records, facilitating data collection, facilitating analysis, facilitating decision making (I.7)
	Hospitals upgrade information systems infrastructure that can accommodate digital transformation changes.	What we need to optimize is to upgrade the hospital SIM from the previous version to a more sophisticated version, a complete version to accommodate the needs of electronic medical records, both outpatient and inpatient care, and also accommodate telemedicine, then create a patient portal (I.4)
Effective leadership (I.1, I.10, I.11, I.12, I.14, I.16, I.17, I.18)	Leaders have the ability to read situations	Of course, from the leadership, the speed of getting information and reading the existing situation, and then identifying existing problems, it seems that we can do anything to implement it in our organization (I.10)
	Leadership has a mindset that every change is an opportunity	(Hospitals must have excellent leadership with good mindset that they could take advantage of this situation and not just survive.) (I.16)
	Leaders who are visionary and able to direct members of the organization to be able to do something	It takes a leader who will be able to drive people in the organization so that they can do it and have an excellent vision to control all of this. (I.11)
	Leaders have communication skills both inside and outside the organization	The director of the hospital must have good communication with the governor, the regional parliament, the budget agency, the Ministry of health, and also below (member of the organization) (I.14)
Flexibility in managing existing resources(I.1, I.6, I.4, I.31.10, I.13,1.15)	Hospitals can reconfigure medical devices to use them for different purposes.	How is the existing equipment, even though it is the same but it can be utilized for different purposes (I.1)
	Hospitals have the ability to redesign rooms to deal with disasters	We have to start redesigning the room if a disaster occurs. So this is the lesson learned from this pandemic: people are increasingly aware of how to adjust the situation in facing a natural or non-natural disaster (I.6)
	Flexibility in managing budgets and implementing business financial management	When we are a COVID-19 referrals hospital, that means if I were in the planning department, this has many consequences. The budget must be allocated for COVID-19, where to put this money because we don’t have the budget to buy so much PPE, no flexibility, we need to be flexible in purchasing and allocating budget, we find this financial management practice if we see hospital as a business(I.11)
	Flexibility in procuring resources (I.15)	Compared to the private sector, they buy twice as much as government hospitals. USG, CT SCAN, if we purchase cash, it might be 6 billion. But because of tenders, it ends up multiplying, and if it’s just that it’s so big, while the finances are limited, how he can find this between operational and other cost.? (I.15)
	The organizational structure accommodates flexibility in coordination	Well, the structure is dynamic, not bureaucratic, I can jump directly to the CEO, I can also go directly to the commissioner, if it’s something urgent. Then if we have to discuss some position in the corporate, although there is no direct line to the director, there is a coordination line (I.10)
Situation awareness and resilience ethos (I.1, I.8, I.9, I.11, I.12, I.16, I.20)	Ability to shift threats into opportunities (1.1,1.11)	The ability to manage threats becomes opportunities, primarily related to patient wishes (I.1)
	There is a teamwork capable of translating government policies into policies that can be operationalized at the hospital level.	There is a need for a team in the hospital that really can read the situation of sudden changes, including in the policies of the Ministry of Health or changes in government policies in general (I.8)
	Learning organizational culture thereby facilitates change management	Yes, a learning organization is an organization that continues to learn, which means it supports change management open-mindedness, and there is always space to find out more information (I.12).
	Have alternative tactical funds to be used in times of crisis	Including finances, do you have a tactical alternative or not? That’s an illustration of resilience; these funds can’t be used except for certain conditions (I.20)
	Having regular briefings to find out the current situation of the organization	Understanding environmental conditions is essential. So what should be today means there is a briefing today (I.20)
	Mechanism in preparing contingency planning	For example, do you have an alternative program or not, so there is a contingency plan (I.20)
Implementation of marketing management (I.2, I.8, I.9, I.6, I.10)	ability to assess market needs and create services according to community needs.	The hospital’s ability to assess market needs and create services according to community needs (I.19)
	Ability to create product diversification to improve patient experience	What’s the difference, but how it is served is valued and treated; that’s what makes the experience comfortable. It will be back again and bring others (I.9)
	Hospitals need to have rebranding capabilities that focus on their center of excellence	Now, the hospital has to rebrand its center of excellence, such as a cardiac, stroke, orthopedic, and urology center. (I.6)
	The hospital has a marketing and branding team.	In the private sector, we do have a division called marketing, which is not limited to public relations to sales. The corporation also has marketing and branding divisions. (I.10)
Networking (I.3, I.8, I.9, I.2, I.1, I.19,1.17))	Collaboration with other hospitals in the use of medical equipment (I.3, I.9)	Now, we have to collaborate because today is the year of collaboration. We have to collaborate in the South Sulawesi region. It is not about who is the strongest but about collaborating with anyone who has the MRI and chemotherapy machines so that everything can work effectively (I.3).
	The hospital has a coordination and collaboration mechanism with other hospitals, especially in determining their excellent service.	The importance of coordination and collaboration between hospitals so that each of them develops centers of excellence that don’t overlap (I.8)
	The hospital cooperates with other hospitals in strengthening services.	We have to be partners with other hospitals, not competitors. It will benefit us if we have much networking, facilitate referral services, and so on, right? We back up each other (I.9)
	The hospital has a patient group that can communicate the hospital’s business environment.	So, it is essential to have a patient organization there. So I frankly aspire to go there for balancing earlier
	The hospital collaborates with Pentahelix (government elements, academics, business actors, the public, and the media) in strengthening disaster literacy.	Our system is also unclear even though Indonesia imagines 3900 more in 1 day, there have been eight disasters. So that’s what we have to strengthen, so how do we enhance it? Yes, of course, apart from the duties of the educational institution, contributions from Pentahelix from the university, industry, government, and then from the community are also needed. (I.19)
	Hospitals work with vendors to accelerate digital transformation changes	Hospitals work with vendors. If they can’t make their facility, try the vendor but based on health facility-based facilities, to think about telemedicine (I.2)
	The hospital cooperates with other hospitals in procuring equipment (consolidated goods purchasing)	Of course, there may be a President’s Law regarding consolidated goodpurchasing. So it’s not forbidden. For example, the Soppeng Hospital and nearby Bone Hospital can join because of consolidation. (I.17)
Disaster anticipation (I.11,I.19)	The hospital understands the potential for disaster in its working area	So he has to know when the first one happens, he has to know what types of disasters, then later, what disasters will potentially occur in the area where the hospital is located (I.19)
	Disaster literacy in all human resources	Many nurses and doctors don’t know how to manage the disaster victims. So he doesn’t know, for example, if this is a disaster like this, what should we do, what facilities should we provide. So why is it like that? Because the disaster literacy is lacking (1.19)
	The infrastructure built takes into account resilience in the event of a disaster.	So, all the building structures are not designed like in Japan, they are not designed. For example, if an earthquake occurs, it can be resilient or sturdy; it means literacy again. So when the hospital was built, there was no check that it was OK because we were in an earthquake area. The hospital must be made to a standard that in the event of a six on the Richter scale shock, the hospital remains intact (I.19).
	Standard Operating Procedure (SOP) when a disaster occurs	At that hospital, the SOP was already implemented very extraordinarily. So when there’s an incident, they have an emergency mechanism like that (1.19)

## Hospital resources readiness in facing digital transformations

The readiness of resources in facing digital transformation included the readiness of human resources in dealing with technological changes and the readiness of information system infrastructure to facilitate decision-making, be updated, be compatible with current technology, especially digital transformation. From the human resources aspect, capacity building of human resources in hospitals is necessary as the digitization of operational activities will be a mandatory policy in all hospital systems in Indonesia. The management should be aware that the digitization will change the workflow. All health workers will adapt to the new way of working, for instances, the ability to adapt with the Electronic Medical Records (EMR) system. The roles of hospital information systems that can collect and transform data into information from decision-making is also important. Besides that, the capacity to upgrade the digital infrastructure is also vital to facilitate interoperability of the hospital information system with the BPJS system, registration, payment information system, integrated medical records, data collection, and services.

## Effective and visionary leadership

Effective leadership includes leaders who are able to read the current situation and see changes as opportunities. This capacity is important to reflect how leaders see the context of every situation and how the organization responds to the changes, whether it is a threat or an opportunity. Besides that, it is necessary to have leaders who have a vision, are able to mobilize members to achieve this vision, and have excellent communication skills. Communication skills would entail building interpersonal relationships with members within and outside the organization, both health and non-health organizations.

## Flexibility in managing existing resources

This domain highlights the importance of flexibility in planning, procuring, and organizing resources. In facing disruptions, the hospital must be flexible in managing finances and procuring resources, for instance, procurement of Personal Protective Equipment (PPE) in facing a sudden significant increase in patient demand during the pandemic. This procurement is out of allocation in financial planning but essential to purchase. Under certain conditions, hospitals also need to be able to procure some medical equipment that is urgently available, redesign rooms, and reconfigure their medical devices for different purposes. This domain also focused on a flexible organizational structure that may enable each manager to coordinate not in a bureaucratic way, such as a director being directly able to discuss with the CEO without the need to go through the hospital board.

### Situation awareness and resilience ethos

Situation awareness includes the ability to read organizational situations to identify what should be done, be aware of environmental conditions, and be prepared for potential risks. Besides that, hospitals must be able to manage every change and challenge. To address the threats, learning organization capacities that allow continuous learning and open-mindedness are also important. Besides that, the hospital has a resilient ethos. This capacity could be reflected in individual or team capacities, mindset, and organizational culture. For instance, at the individual or group level, the hospital has teamwork that can learn and operationalize government policy changes into a hospital regulation mindset in doing simple research for hospital innovation. At the organizational level, the hospital has tactical funds that can only be used in times of crisis and a habit of conducting regular briefings to identify the current conditions and even contingency plan mechanisms within the organization.

### Implementation of marketing management

This domain represents marketing management functioning in hospitals. In Indonesia, marketing management is still shown as a very modern approach in the transition era of hospitals seen as a social organization to industry managed as a business. Some informants highlighted that the function of marketing management is important. This includes the hospital’s ability to conduct simple research based on patient characteristic data, assess market needs and create services according to community needs, diversify products to improve patient experience, rebranding capabilities for focus on centers of excellence, and have a team to apply marketing and branding function. Besides that, implementing marketing management in hospitals could assist hospitals in finding their unique, excellent value and their centers of excellence.

## Networking

Networking capabilities include how hospitals have a collaborative network with other hospitals, for example, collaboration in hospital resources procurement, collaboration in the use of medical equipment, discussions in determining centers of excellence in each hospital. Networking with non-health organizations would also be beneficial and could include collaborating with the government, academics, business actors, the community, media, and patient groups.

## Disaster anticipation

Indonesia is located in a disaster-prone area. It is crucial to have the ability to understand potential disaster risks in work areas and human resources should be exposed to disaster literacy. Hence, they understand what to do when a disaster occurs, prepare resilient hospital infrastructure that can withstand a disaster, and have disaster-related standard operational procedures.

## Discussion

We have presented seven domains for resilient hospitals in facing a disruption era. The domains highlighted the capacities hospitals should have in order to face the disruptions era which is characterized by a rapidly evolving environment that requires capacities to adapt to any changes in all given periods. These domains contrast the past hospital resilience concept, which mainly focused on the hospital’s ability to deal with specific shocks, such as disasters [11, 21, 22, 51, 90]. However, hospital resilience when facing disruptions demonstrates shocks from different perspectives. The changes resulting from disruptions are inevitable within the organizational business environment, which is not categorized in the specific shock and, therefore, not generally discussed in the Hospital Disaster Resilience (HDR) concept [10, 11].

One of the domains that stands out is the readiness to face digital transformation. This domain highlighted specific conditions encountered in the disruption era characterized by digital disruption technology. Several trends in the development of digital transformation that affect health facilities are the use of big data in health services, treatment of patients with Virtual Reality (VR) and Augmented Reality (AR) technology, wearable medical devices, blockchain, and electronic medical records [91]. Digitization makes healthcare less expensive and enables the recreation of healthcare services to become more agile and responsive to technological changes [92]. In Indonesia, the Ministry of Health initiated a digital transformation policy that is made mandatory to all hospitals in 2023. These policies cause shifting in the operational level system as it becomes a mandatory requirement for hospitals to officially join the Social Security Administration Agency (BPJS). Consequently, each hospital must adjust its information system and all resources to prepare the BPJS bridging system.

From the seven domains identified in our study, three domains have been identified in previous health system resilience studies such as leadership [3, 15, 93, 94], flexibility in managing existing resources [95], and networking [3, 15]. Our findings also show that leadership is an essential domain for resilience [67, 96, 97]. The capacities of leaders highlighted in this study focused on envisioning the current situation, empowering others, and having communication skills. Lying et al. (2022) also mentioned these leadership capacities as prioritizing, empowerment, and interaction. Leaders’ ability to read situations will influence the capacity to prioritize strategies based on demands and organizational capacities. This shows the contextual understanding of leaders that affects how leaders adapt and respond in every situation. Empowerment capacity is also shown as encouraging staff to take on responsibilities. The interaction capacities mentioned by Lyeng et al. (2020) focus on the importance of providing support and motivation to staff; good communication skills are necessary to have this capacity.

Another domain identified is situation awareness and resilience ethos. This domain is developed in the organizational resilience concept in management studies [67, 68]. Situation awareness implies the capacity to understand the situation around and be aware of its implication to the current and future position of the organization [54, 67]. Another study revealed this situation awareness capacity as the cognitive resilience of an organization [63]. For example, Lengnick-Hall (2011) describes situation awareness capacities as a genuine orientation that allows the interpretation of unprecedented events and conditions of the organization.

As a result of societal changes, hospitals face changes concerning customer needs and lifestyles that influence patient expectations, hospital revenue, and the hospital market [98]. Therefore, hospitals have to develop new approaches to hospital management, such as marketing management and creating centers of excellence. Marketing management is vital in creating value by understanding consumer needs and expectations, giving the hospital a competitive advantage, and building a strong brand image in the hospital services market [99]. In Indonesia, marketing management is a new function in hospital management. This function seems to be used more in private institutions than in government sectors. The findings of this study highlight the importance of marketing management functions and having centers of excellence in order to enhance hospital capacities to be resilient in Indonesia.

Finally, this study found one domain that focuses on disasters, namely disaster anticipation. This is due to Indonesia’s condition, which is a disaster-prone area for earthquakes, tsunamis, volcanic eruptions, floods, forest fires, droughts, landslides, and flash floods [100, 101]. The concept of disaster in this study focuses on the ability of hospitals to identify disaster risks in their area, establish standard operating procedures in disaster events, and increase disaster literacy in hospital human resources. This disaster concept is aligned with the pillars of disaster resilience developed by the National Planning Development Agency (2015); every institution must understand the concepts of risk identification, risk reduction, preparation, financial protection, and resilient recovery.

However, this study has some limitations. As part of the Conceptual Model of Hospital Resilience Study in Indonesia, informants such as policymakers, hospital practitioners, and academicians involved are only from Indonesia. The capacity of hospital resilience could be specific based on the context of disruption faced by the hospital Industry in Indonesia. The domain of hospital resilience in the context of the global hospital industries in the disruption era could be highlighted if the informants also involved collaborators to gain a global perspective. Besides, this exploratory study could not show which domain significantly contributes to hospital resilience. Therefore, future studies need to show the influence of each domain on hospital resilience and how each domain correlates.

## Conclusion

This paper addresses comprehensive hospital resilience domains and indicators that do not only focus on disaster aspects but also the disruptions faced by hospitals. The domain of hospital resilience in the era of disruption is more to prepare hospitals to face digitalization and compete with the market through centers of excellence, organizational structure flexibility, effective leadership, and the ability to redesign resources. In addition, in the context of a continuously changing hospital environment, hospitals need to have situation awareness capabilities, implement marketing management, expand networking, manage hospitals with modern management, and remain prepared in the face of disasters. This domain is a resilience capability that is developed according to experts from the perspective of the hospital context in Indonesia. The results of this qualitative research need to be continued for future research by verifying the construct indicators for the domains that have been identified. These findings give us insight into how resilience could be operationalized at the level of health facilities and could be used as self-assessment tools for hospitals to determine the level of hospital resilience.

### Supplementary Information

**Additional file 1.**

## Data Availability

The datasets generated and analyzed in this study are available from the corresponding author upon reasonable request.

## Abbreviations

VUCA: Volatility, Uncertainty, Complexity, Ambiguity
EMR: Electronic Medical Records
HSI: Hospital Safety Index
HDR: Hospital Disaster Resilience
AI: Artificial Intellilgent
