# Supplementary material for: Developing hospital resilience domains in facing disruption era in Indonesia: a qualitative study
Source: BMC Health Serv Res. 2023 Dec 12;23:1395. doi: 10.1186/s12913-023-10416-8 (PMC10714512; doi:10.1186/s12913-023-10416-8)
Supplement: Supplementary file 1 — Additional file 1. [file 12913_2023_10416_MOESM1_ESM.docx]

**Supplementary File 1**

**INTERVIEW GUIDELINE**

Assalamu Alaikum Wr. Wb / Shalom / Good morning/afternoon, Madam.

Thank you for your interest in participating in the research titled “Conceptual Model and Measurement of Hospital Organizational Resilience in the Era of Disruption in Indonesia.” In general, this research aims to develop a conceptual model and measurement of hospital organizational resilience in the era of disruption in Indonesia. This research will go through several stages: 1) exploring the disruptions faced by hospitals in the context of the hospital industry in Indonesia; 2) developing dimensions and indicators of hospital organizational resilience.

In the healthcare system, hospitals play a vital role in disasters and are expected to have good levels of resilience to continue functioning optimally and supporting community resilience. In its application, the concept of hospital resilience has been developed by the World Health Organization (WHO), the Pan American Health Organization (PAHO), and several researchers, particularly in disaster-related areas such as climate, earthquakes, floods, and pandemics. This concept focuses more on identifying resources needed due to direct disaster-related damages, such as infrastructure, emergency service coordination, and various aspects of disaster preparedness. However, with the increasing challenges from external factors such as technological changes, financial crises, policy changes, and multiple threats faced by hospitals today, this concept of resilience has not accommodated challenges beyond the scope of disaster issues. Despite the characteristics of organizations, hospitals, with all their complexities, uncertainties, externalities, and high risks, will continue to face various internal and external challenges. The resilience in this research aims to explore the capacity hospitals should have in facing the dynamic environment in the era of disruption.

Phase 1

1. According to Shwan & Crisson (2020), there are several changes in healthcare services in the era of disruption, including technological, economic, and social changes.

a. In your opinion, what are the technological disruptions or disruptions in hospitals currently?

b. In your opinion, what are the economic disruptions or disruptions in hospitals currently?

c. In your opinion, what are the social disruptions or disruptions in hospitals currently?

1. Besides these three disruptions, what other disruptions or disruptions are there in hospitals currently?

Phase 2

1. How should hospitals deal with these disruptions or disruptions? (This question is based on their responses to the shocks faced by hospitals mentioned in stage 1)
